# Antibacterial Activity of *Barringtonia acutangula* against Selected Urinary Tract Pathogens

**DOI:** 10.4103/0250-474X.45417

**Published:** 2008

**Authors:** S. Sahoo, P. K. Panda, S. R. Mishra, R. K. Parida, P. Ellaiah, S. K. Dash

**Affiliations:** University Department of Pharmaceutical Sciences, Utkal University, Bhubaneswar-751 004, India; 1 Pharmaceutical Biotechnological Division, Department of Pharmaceutical Sciences, Andhra University, Visakhapatanam-530 003, India; 2 P. G. Department of Microbiology, Orissa University of Agriculture and Technology, Bhubaneswar-751 003, India

**Keywords:** *Barringtonia acutangula*, antibacterial activity, UTI pathogens, minimum inhibitory concentration

## Abstract

*Barringtonia acutangula* (L.) Gaertn belonging to family Barringtoniaceae was investigated to evaluate *In vitro* antibacterial activity of aqueous, ethanolic, petroleum ether and chloroform extracts against *Staphylococcus aureus*, *Pseudomonas aeruginosa, Klebsiella pneumoniae, Enterococcus faecalis* and *Escherichia coli* the major urinary tract infection causing pathogens were tested by disc diffusion assay method and the minimum inhibitory concentration was evaluated. Ethanol (95%) extract exhibited broader spectrum of inhibition followed by chloroform, petroleum ether and aqueous extracts against the urinary tract pathogens under test. An attempt has been made to compare the activity of extracts with standard antibiotics against selected urinary tract infection causing pathogens.

*Barringtonia acutangula* (L.) Gaertn belonging to family Barringtoniaceae is a medium size glabrous tree found throughout India in decidous and evergreen forests, mostly along the bank of rivers and streams^1^. It is used in the folklore in vitiated conditions of *kapha* and *pitta*, leprosy, arthralgia, dysmenorrhea, plumbago, skin diseases, diarrhea, inflammation, flatulence, hemorrhoids, as an anthelmintic^2^. The plant has been reported to have antiimplantation activity in female albino rats^3^. In Ayurveda, its preparations include powder and pastes. The present study is intended to determine the antibacterial activity of the plant against selected urinary tract pathogens by disc diffusion assay^4^,^5^.

The plant material (twig) was collected from Keonjhar district of Orissa and authentificated by the taxonomist of Department of Botany, Utkal University, Bhubaneswar. One voucher specimen (No. UDB/B-912) was deposited in the herbarium of the department for future reference. After authentification the seeds were collected in bulk, shade dried for 2 d and then dried in the hot air oven at 50° for 12 h. Dried seeds were pulverized by a mechanical grinder and the coarse powder obtained was taken for extraction in petroleum ether followed by chloroform, ethanol (95%) and water by using Soxhlet assembly for 48 h each. The extracts were dried under reduced pressure and the percentage of yield was calculated on the dried weight of extract. The *in vitro* screening for antimicrobial was carried out using selected urinary tract infection (UTI) causing pathogens which includes two gram positive bacteria *(Staphylococcus aureus* and *Enterococcus faecalis)* and three gram negative bacteria *(Escherichia coli, Pseudomonas aeruginosa* and *Klebsiella pneumoniae)*. Five strains each of the 5 UTI causing bacterial species were used in this study were obtained from Post Graduate Department of Microbiology, Orissa University of Agriculture and Technology, Bhubaneswar, Orissa. The antibacterial screening of the extracts were carried out by determining the zone of inhibition using disc diffusion method^4^,^5^. The extracts dissolved in dimethylformamide (DMF) at a concentration of 50 mg/ml and finally sterilized by filtration using 0.45 μm Millipore filters. The sterile discs (6 mm in diameter) were impregnated with 2.5 and 20 μl of above extract solution to achieve desired concentration of 125 and 1000 μg/disc and placed in inoculated agar. The density of the bacterial suspension was standardized by standard McFarland method^6^. Amoxicillin+clavulinic acid (AC) (20+10) (30 μg/disc) and ciprofloxacin (CF) (25 μg/disc) were used as standards. The inoculated plates with the test and standard discs on them were incubated at 37° for 24 h. The zone of inhibition of different extracts and standard drugs by disc diffusion method and an attempt has also been made to compare the antibacterial activity of *B. acutangula* extracts with the potent standards effective against the selective UTI pathogens is given in Table 1. The minimum inhibitory concentrations (MIC) of the extracts were determined by using two fold serial dilution assay^7^ for the microorganisms which were determined sensitive to various extracts of *B. acutangula* in disc diffusion assay. The petroleum ether, chloroform, ethanolic and aqueous extracts were dissolved in mixture of 6% DMF and then diluted to the highest concentration (1000 μg/ml) subsequently two fold serial dilutions were made in a concentration range from 7.8-1000 μg/ml. MIC values of the extracts against UTI pathogens were determined with some modifications. The dilutions were performed by dispensing into each tube 1 ml of nutrient broth and 1ml of each extract to achieve concentration of 1000 μg/ml and then serially diluted. Fifty microlitres of freshly prepared inoculum was then added to all the tubes including control and incubated at 37° for determination of MIC. The MIC values of the extracts were determined by two fold serial dilution assay are given in Table 2.

**TABLE 1:** *IN VITRO* ANTIBACTERIAL ACTIVITY OF *BARRINGTONIA ACUTANGULA* EXTRACTS AGAINST URINARYTRACT PATHOGENS BY DISC DIFFUSION METHOD

Org.	Extract Conc. (μg/disc)	Zone of Inhibition (in mm)*					

		PE	CH	ET	AQ	CF	AC
1	A	18.3±0.50	21.2±0.55	22.4±0.55	17.2±0.65	25.3±0.15	25.3±0.35
	B	11.4±0.45	18.3±0.50	21.3±0.65	11.9±0.5		
2	A	17.2±0.7	16.8±0.45	17.5±0.4	19.8±0.90	26.4±0.45	12.0±0.40
	B	12.8±0.90	11.7±0.55	14.4±0.66	10.5±0.66		
3	A	18.5±0.66	19.3±0.65	17.1±0.70	11.7±0.55	25.4±0.35	17.1±0.36
	B	13.5±0.65	17.4±0.70	13.8±0.70	9.4±0.55		
4	A	18.3±0.55	17.2±0.70	23.5±0.75	17.2±0.6	26.4±0.45	10.7±0.70
	B	12.3±0.80	16.6±0.90	17.2±0.60	12.4±0.65		
5	A	13.5±0.75	12.5±0.66	19.2±0.75	19.3±0.75	28.0±0.15	-
	B	10.1±0.45	12±0.70	16.5±0.7	13.4±0.60		

- Indicates no zone of inhibition. A and B indicates the concentrations of the extracts at 1000 and 125 μg/disc, respectively. Org-organisms 1. *Staphylococcus aureus*; 2. *Pseudomonas aeruginosa*; 3. *Klebsiella pneumoniae*; 4. *Enterococcus faecalis*; 5. *Escherichia coli*; PE, CH, ET and AQ stands for petroleum, chloroform ether, ethanol (95%) and aqueous extracts, respectively. CF and AC stand for ciprofloxacin 25 μg/disc and amoxycillin+clavulinic acid (20+10) μg/disc, respectively.

* All the values are mean±standard deviation of three determinations

**TABLE 2:** THE MIC VALUES OF EXTRACTS AGAINST THE MICROORGANISMS TESTED BY TWO FOLD SERIAL DILUTION ASSAY.

Microorganisms	MIC			
	
	Pet ether	Chloroform	Ethanol	Aqueous
*S. aureus*	250	62.5	62.5	500
*P. aeruginosa*	250	250	125	250
*K. pneumoniae*	500	125	250	1000
*E. faecalis*	250	125	62.5	250
*E. coli*	500	500	125	250

MIC is minimum inhibitory concentration in μg/ml.

All extracts of *B. acutangula* at 1000 μg/disc showed optimum activity against all tested UTI pathogens. However, results of disc diffusion method as indicated in Table 1 revealed that ethanolic extract showed highest activity followed by chloroform, petroleum ether and aqueous extracts against the UTI pathogens under test. Ethanol extract showed highest inhibition (23.5±0.75 mm) and lowest (17.1±0.70 mm) against *E. faecalis* and *K. pneumoniae* at 1000 and 125 μg/disc, respectively. Chloroform extract showed highest inhibition (21.2±0.55 mm) and lowest (11.7±0.55 mm) against *S. aureus* and *P. aeruginosa* at 1000 and 125 μg/disc, respectively. Petroleum ether showed highest inhibition (18.5±0.66 mm) and lowest (10.1±0.45 mm) against *K. pneumoniae* and *E. coli* at 1000 and 125 μg/disc, respectively. Aqueous extract showed highest inhibition (19.8±0.90 mm) and lowest (9.4±0.55 mm) against *P. aeruginosa* and *K. pneumoniae* at 1000 and 125 μg/disc, respectively. The zone of inhibition exhibited by the extracts of *B. acutangula* at 1000 μg/disc exhibited comparatively equivalent activity to that of standards CF 25 μg/disc and AC (20+10) μg/disc. MIC of *B. acutangula* extracts against the tested UTI pathogens as indicated in Table 2 showed that ethanol and chloroform extracts inhibited microbial growth at comparative lesser concentration to that of petroleum ether and aqueous extracts. Further studies aimed at isolation and characterization of active phytoconstituents from extracts with antibacterial potential.
